# Microbial Biotransformation of Agro-Industrial Fibre-Rich By-Products into Functional Beverages

**DOI:** 10.3390/antiox14111332

**Published:** 2025-11-05

**Authors:** Pau Sentís-Moré, Ivan Robles-Rodríguez, Kevin Leonard, Job Tchoumtchoua, Xavier Escoté-Miró, Josep M. del Bas-Prior, Nàdia Ortega-Olivé

**Affiliations:** 1 Unitat de Nutrició i Salut, Eurecat—Centre Tecnològic de Catalunya, 43204 Reus, Spain; pau.sentis@eurecat.org (P.S.-M.); xavier.escote@urv.cat (X.E.-M.); josepm.delbas@urv.cat (J.M.d.B.-P.); 2 Department of Food Technology, Engineering and Science, University of Lleida, 25198 Lleida, Spain; 3 Biomass Valorization Platform, CELABOR Srl, 4650 Herve, Belgium; kevin.leonard@celabor.be (K.L.); job.tchoumtchoua@celabor.be (J.T.); 4 Center of Environmental, Food and Toxicological Technology (TecnATox), University Rovira i Virgili, 43007 Tarragona, Spain; 5 Nutrition and Metabolic Health Research Group, Department of Biochemistry and Biotechnology, Institute of Health Pere Virgili (IISPV), Universitat Rovira i Virgili, 43007 Tarragona, Spain; 6 Nutrigenomics Research Group, Department of Biochemistry and Biotechnology, Universitat Rovira i Virgili, 43007 Tarragona, Spain; 7 Agrotecnio-CERCA-Centre, 25198 Lleida, Spain

**Keywords:** lactic acid fermentation, fermented beverages, by-products, fibre-rich by-products, *Lactiplantibacillus plantarum*, *Bacillus subtilis*, antioxidants, functional foods

## Abstract

This study explores the development of functional fermented beverages using fibre-rich residues derived from olive pruning, vineyard pruning, chicory root, and red onion, obtained after subcritical water extraction of polyphenols. Two microbial strains, *Lactiplantibacillus plantarum and Bacillus subtilis,* were evaluated for their fermentation performance across different fibre matrices, with and without sugar supplementation. Key parameters including microbial growth, pH evolution, and reducing sugar content were monitored, and *Lactiplantibacillus plantarum* showed superior acidification and viability (>9 log CFU/mL), especially in sugar-enriched formulations, while *Bacillus subtilis* showed a limited performance. Based on fermentation efficiency, three sugar-supplemented formulations were selected to scale-up: olive pruning fibre and vineyard pruning fibre fermented with *Lactiplantibacillus plantarum* and olive pruning fibre fermented with *Bacillus subtilis*. Red onion fibre extract was excluded from scale-up experiments due to its high viscosity, which made it impossible to measure reducing sugars, consistent with its high water-holding capacity. Fermentation significantly increased antioxidant capacity, reaching up to 750 µmol Trolox equivalents/L and 18 mg of gallic acid equivalents/L in *L. plantarum*-fermented samples, confirming microbial release of bound phenolics and formation of bioactive metabolites. The resulting beverages were microbiologically stable (final pH < 4.5), sensorially acceptable, and potentially antioxidant-rich, supporting their role in sustainable food system development and circular bioeconomy.

## 1. Introduction

Food waste and agro-industrial by-products are generated in substantial quantities worldwide, with the European Union alone producing approximately 88 million tons annually, which represents a financial loss of over EUR 143 billion [1]. Traditionally, these residues have been repurposed as animal feed or compost. However, current sustainability goals, particularly those aligned with the European circular economy framework, emphasise the valorisation of such materials into higher-value products. Notably, these by-products are often rich in dietary fibres, polyphenols, and other bioactive compounds with proven health-promoting potential [2,3].

Fermentation is a time-honoured food preservation and transformation process that leverages microbial enzymatic activity to convert complex substrates into simpler, often bioactive molecules. Submerged fermentation—carried out in aqueous media—is particularly suitable for producing functional fermented beverages. These beverages have garnered growing attention for their potential health benefits, particularly through modulation of the gut microbiota and enhancement of bioactive metabolite production [4,5].

Agro-industrial by-products offer an abundant and underexplored substrate for such applications. Fermentation using lactic acid bacteria (LAB), yeasts, and selected fungi has been shown to enhance the nutritional and functional properties of these materials, creating value-added products with benefits for sustainability. LAB strains, such as *Lactiplantibacillus plantarum (Lp)* and *Pediococcus pentosaceus (Pp)*, are especially effective due to their ability to ferment dietary fibres and produce lactic acid, which acts as a natural preservative while improving organoleptic qualities [6]. In addition, *Bacillus subtilis* has been shown to efficiently convert insoluble dietary fibre into soluble dietary fibre, enhancing both the structural and functional properties of fibre-rich residues. This strain exhibits high cellulase activity and maintains robust growth in fibrous substrates, making it a particularly promising microorganism for the biotransformation of agro-industrial by-products into functional and value-added ingredients [7]. For instance, fermentation of Prosomillet bran with LAB increased the yield of soluble dietary fibre from 4.2% to 7.6%, modified the microstructure to become more porous, and improved physicochemical and functional properties such as solubility, water, and oil-holding capacity, swelling capacity, and adsorption of nitrite, sodium cholate, and cholesterol. Moreover, phenolic content and antioxidant activity were significantly enhanced, demonstrating the potential of fermented soluble dietary fibre as a functional food ingredient [8]. A variety of fermented functional beverages, such as kefir and kombucha, which demonstrate immunomodulatory and antimicrobial benefits, already exist globally [9]. Market interest in fermented non-alcoholic beverages with high nutritional value and palatable sensory profiles has stimulated innovation using plant-based materials and food by-products [10,11]. Fruit peels, pomace, and vegetable trimmings, among others, are being investigated for their polyphenol content, prebiotic potential, and fermentability [12].

Despite these advances, comprehensive studies focusing on the fermentation of fibre-rich extracts—particularly those recovered after polyphenol extraction—remain scarce. This research gap is largely due to the intrinsic challenges of fermenting high-fibre matrices. Fibre-rich residues typically contain low levels of readily fermentable carbohydrates, which can limit microbial growth and acidification rates. In addition, the structural complexity and high water-holding capacity of insoluble fibres can hinder the diffusion of nutrients and metabolites, reducing the efficiency of the fermentation [4,13]. Furthermore, residual phenolic compounds—though beneficial for health—may exhibit antimicrobial activity that interferes with microbial proliferation [14]. As a result, successful fermentation of these materials often requires process optimisation, such as carbon source supplementation or selection of robust microbial strains.

Nevertheless, fermenting fibre-rich by-products offers distinct advantages. Even after polyphenol extraction, these matrices can retain functional polysaccharides (e.g., pectins, fructans, arabinoxylans) and bound phenolics that are substrates for microbial enzymatic conversion into bioactive metabolites [2,3]. Such biotransformations have been linked to enhanced antioxidant capacity, modulation of gut microbiota, and improved sensory characteristics [5,15]. These benefits make the valorisation of fibre-rich residues an attractive strategy within models of the circular economy, provided hurdles to fermentation can be overcome through targeted technological interventions.

This study aimed to develop functional fermented beverages from fibre extracts obtained from four agro-industrial by-products: olive pruning fibres (OPFs), vineyard pruning fibres (VPFs), chicory root fibres (CRFs), and red onion fibres (ROFs). These fibres were recovered following subcritical water extraction of polyphenols, ensuring minimal residual phenolics and a high fibre content. We evaluated the fermentability of these substrates using two microbial strains—*Lactiplantibacillus plantarum (Lp)* and *Bacillus subtilis (Bs)* —with and without sugar supplementation. This study monitored microbial growth, pH evolution, and reducing sugar dynamics across conditions. Ultimately, this work supports the design of sustainable, scalable, and microbiologically safe fermented beverages, thus demonstrating a practical pathway for valorising food system by-products and promoting environmental sustainability.

## 2. Materials and Methods

### 2.1. Plant Materials

Chicory roots (CRs), *Cichorium intybus* var. *foliosum,* were provided by Potager St Germain (Soiron, Belgium). Olive prunings (OPs), *Olea europaea* var. *verdale aglandau*, were collected by Saint André GAEC (Merschweiller, France) on 25-year-old trees. Vine prunings (VPs), *Vitis vinifera* var. *caberbnet franc,* came from 7-year-old trees of Chateau de la Martinette SCEA (Lorgues, France). Both olive and vine samples were shipped to Celabor by Chambre Régionale d’Agriculture Provence Alpes Côte d’Azur. Cartif Technology Centre (Valladolid, Spain) arranged the shipment of red onion peels (RO), *Allium cepa*, coming from Casilla-la-Mancha (Spain).

### 2.2. Recovery of the Remaining Fibre Fraction After Extraction of Polyphenols by Subcritical Water Extraction

**Figure 1:**
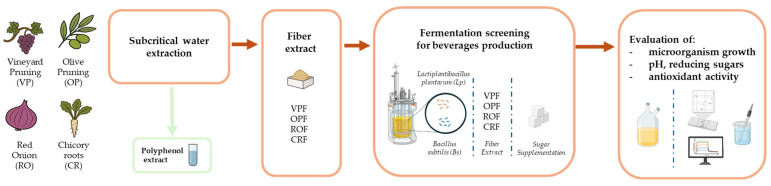
Graphical representation of the recovery of fibre fractions after subcritical water extraction of polyphenol (CRF, OPF, VPF, and ROF) and their use as raw materials for fermented beverage development.

Fibre extracts were produced from the four feedstocks (CR, OP, VP, and RO) after extraction of polyphenols by subcritical water extraction: CRF, OPF, VPF, ROF (Figure 1). Dried raw materials were milled (2–4 mm sieve) and dropped into a six-litre stainless-steel insert. The insert was introduced into a pre-heated reactor and the system was closed. Subcritical water extraction was carried out at 120 °C for CRP and ROF residues, and at 150 °C for OPF and VPF, under 15 bar for 30 min with a flow rate of 1000 g/min. Automatic valves were closed and the recirculation pump was switched to recirculate the water in the extraction loop at a flow rate of 1000 g/min. Recirculation was maintained for 30 min. Then, the liquid extract was cooled prior to depressurising the system. The total liquid extract was flushed into the collector using a nitrogen flow to drain the system. Two complete cycles of extraction were performed. The global extract was collected from the separator. The extracted raw material rich in fibre was recovered from the stainless-steel insert and dried at 45 °C for 48 h.

### 2.3. Material Characterisation

To evaluate the adequacy of the samples for the fermentation process, the humidity, fibre composition, and water-holding capacity (WHC) of samples were evaluated. The measurements were performed in duplicate.

#### 2.3.1. Moisture Content

Moisture content was determined with the oven drying method, where 5 g of the sample were placed in an oven for 4 h at a constant temperature of 105 °C until a constant weight was reached. The moisture content was calculated with through the weight difference in the sample, expressed in g/100 g.

#### 2.3.2. Water-Holding Capacity (WHC)

The measurement of the WHC was adapted from [16,17]. Briefly, 5 g of sample was diluted in water and mixed for 1 h in an orbital shaker. The mix was centrifuged for 15 min, and the supernatant water was discarded. The water-holding capacity was calculated from the difference in weight from the initial sample considering its humidity, and expressed according to the following equation:

(1) $$Water\;Holding\;Capacity\;\left(WHC\right)\;\left(\%\right)=\;\left(\left(\frac{Final\;weight\;(g)}{Initial\;weight\;(g)}-1\right)\;\times \;\frac{86}{100-Moisture\;(\%)}\right)\;$$

### 2.4. Production of Fermented Beverages

The procedure followed to produce fermented beverages consisted of two different workflows, including the propagation of the inoculum and the procedure of the beverages.

#### 2.4.1. Propagation of the Inoculum

The microorganisms used in the present study were acquired from ATCC: *Lactiplantibacillus plantarum (Lp)* (ATCC 14917) and *Bacillus subtilis* (*Bs*) (ATCC 15245). Both microorganisms were activated with the handling information from the supplier. The Man–Rogosa–Sharpe Broth (MRS) medium growth (ThermoFischer Scientific, Waltham, MA, USA) was used for *Lp* with a growth condition of 30 °C in an aerobic atmosphere. A nutrient broth culture medium (Sigma-Aldrich, St. Louis, MO, USA) was used for *Bs* with a growth temperature of 30 °C.

To propagate the inoculum, 1 mL was inoculated in 10 mL of medium broth under the respective growth conditions for 24 h. After, the inoculum was centrifuged at 8000 rpm for 5 min at 4 °C. The supernatant was discarded, and the pellet was resuspended and diluted with sterile water until an optical density of 0.6 was achieved, using a spectrophotometer at 600 nm (OD600).

#### 2.4.2. Procedure of Screening Experiments

The procedure was adapted from previously described methods [9,18]. Briefly, the samples were ground using an M20 Universal mill (IKA, Staufen, Germany) and sieved at 0.50 mm to homogenise the particle size. Two different formulations were prepared for each fibre extract sample, each containing 10 g of dry matter and 100 mL of filtered water, through a 0.22 µm membrane filter. In one of the formulations, 10 g/L of table sugar was added. Samples were then homogenised using sterile spatulas under aseptic conditions and pasteurised for 30 min at 65 °C to avoid undesired growth of microorganism. After cooling at room temperature, the samples were inoculated in sterile conditions with 1% of the inoculum preparation, either *Lp* or *Bs,* corresponding to an initial microbial concentration of 5.75 log CFU/mL or 5.98 log CFU/mL, respectively. Fermentation was carried out in two phases. In the first phase, the prepared samples were fermented for 72 h in a stove at 30 °C. Following this, the fermented mixtures were filtered under sterile conditions using a Büchner funnel with a vacuum pump and a super-fast filtration paper filter of quality 600 (Dorsan^®^). The retained solid was discarded, and the filtrate liquid was transferred to sterile bottles. In the second phase, the filtered beverages were subjected to an additional 72 h of fermentation at 30 °C. After this period (total fermentation time of 144 h), the samples were bottled and stored at 4 °C until further analysis. The experimental design and sample nomenclature are presented in Table 1.

#### 2.4.3. Procedure of Scaled Experiments

After the screening experiments, conditions were selected based on the fermentation parameters of pH, microbial growth, and sensorial evaluation.

The procedure followed in the scaled experiments was identical to that in the screening experiments, increasing the scale to 1:5, with 50 g of sample, maintaining the proportions of water, sugar, and inoculum.

### 2.5. Fermented Beverage Monitoring

Fermentation was monitored in the initial and final stages of both fermentation periods with the determination of pH, reducing sugars, and the count of viable microorganisms.

#### 2.5.1. pH

pH was determined by the TESTO 205 portable pH meter (Instrumentos Testo, Cabrils, Spain).

#### 2.5.2. Count of Viable Microorganisms

To assess the concentration of microorganisms, viable microorganisms were counted. Briefly, a serial dilution bank was made with PBS, and 0.1 mL of every dilution was pipetted into the Petri dishes. The medium growth of MRS broth was used for *Lp,* and the nutrient broth medium growth was used for *Bs*. European bacteriological agar (Condalab, Madrid, Spain) was added to the broths to make the medium culture plates. Both were incubated in a stove at 30 °C and counted after 24 h. The following formula was used to obtain the total microorganism count, expressed as log CFU/mL:

(2) $$Viable\;microorganism\;count\left(\frac{\log CFU}{mL}\right)=\log\left(\frac{\Sigma \;two\;consecutive\;dilutions}{Inoculation\;volume\;\times \;(1+0.1)}\right)\times \;Number\;of\;dilution$$

#### 2.5.3. Reducing Sugar Content

Reducing sugar content was assessed with by the Rebelein method, using a reducing sugar analysis kit (GAB Sistemática Analítica S.L., Barcelona, Spain). Briefly, the determination uses a titration of the residual copper obtained from the reduction of the sugars in the sample. Results were expressed in g/L.

#### 2.5.4. Total Polyphenol Content

Total polyphenol content was determined according to the Folin−Ciocalteu colorimetric method. An aliquot of 1 mL of fermented beverage was diluted to 25 mL of Milli-Q water, and 2.5 mL of Folin−Ciocalteu reagent was added. After, 5 mL of sodium carbonate was added, and the content was mixed to obtain a final volume of 50 mL with Milli-Q water. After 60 min of standing in darkness, the colour was measured in the spectrophotometer at 750 nm. Gallic acid was used as a standard (concentration range from 0 to 1000 mg/L) with Milli-Q water as a blank. The calibration curve was obtained by linear regression, and results were expressed in mg of gallic acid equivalents (GAE)/L.

#### 2.5.5. Antioxidant Activity

The antioxidant activity was performed by an ORAC assay as described by Ou et al. [19], slightly modified, in a FLUORstar optima spectrofluorometer (BMG GmbH, Offenburg, Germany). The reaction was carried out in phosphate buffer (pH 7.4). AAPH was used as a peroxyl generator (prepared in assay buffer at 37 °C immediately before each assay), with Trolox as a standard (concentration range from 10 to 100 μM) and assay buffer as a blank. The ORAC values were calculated by a linear regression equation between Trolox or the sample concentration and the net area under the FL decay curve. The results are expressed as micromoles of Trolox equivalent per L of fermented beverage.

#### 2.5.6. Sensory Evaluation—Preliminary Screening

A preliminary sensory screening analysis was conducted using a focus group consisting of 6 participants who were experienced in fermented beverages. The objective was strictly to identify major sensory defects or undesirable characteristics in the formulations. Participants assessed key attributes such as aroma and flavour, specifically to detect off-notes or other significant negative traits. Only qualitative feedback was collected and used for quality screening.

### 2.6. Statistical Analysis

The data are presented as the mean ± standard deviation of at least two replicates. The statistical analysis of the full factorial was carried out using JMP^®^ Pro 16.0.0 (SAS Institute, Cary, NC, USA). The results were evaluated using one-way analysis of variance (ANOVA) and Tukey’s test at a 0.05 significance level.

## 3. Results

### 3.1. Physicochemical Characterisation of Fibre Extract By-Products

The initial characterisation of the fibre-rich extracts obtained from OPF, VPF, CRF, and ROF was conducted to assess their suitability for fermentation. The key parameters evaluated included moisture content, fibre composition (soluble and insoluble), and water-holding capacity (WHC). These are summarised in Table 2.

The moisture content across the samples ranged from 4.35 to 8.06 g/100 g, with ROF and OPF exhibiting the highest values. Despite similar moisture levels, significant differences were observed in WHC. This is a critical factor influencing the availability of free water during fermentation. ROF demonstrated the highest WHC (456.65%), followed by VPF and CRF, both exceeding 350%. In contrast, OPF showed the lowest WHC (285.43%), suggesting a lower capacity to retain water.

The analysis of the fibre composition revealed that all sources were predominantly composed of insoluble fibre. VPF had the highest insoluble fibre content (90.5%), while CRF had the lowest (55.9%). Soluble fibre content was generally low across all samples, with OPF containing the highest proportion (2.7%).

A positive correlation was observed between the WHC and insoluble fibre content. Samples with higher insoluble fibre levels, such as VPF and ROF, also exhibited greater WHC. Conversely, OPF, despite its higher soluble fibre content, showed the lowest WHC. These findings suggest that insoluble fibre plays a more significant role in water retention, likely due to its structural properties. However, other factors such as fibre morphology and source-specific fibre-matrix characteristics may also contribute to the variability in WHC.

ROF may contain specific polysaccharides, such as pectins or fructans, with high WHC, or possess a more porous and hydrophilic fibre structure. Additionally, interactions between residual phenolic compounds and fibre components could enhance water retention. These findings highlight the importance of considering both quantitative and qualitative aspects of fibre when evaluating functional properties.

#### 3.1.1. Production of Fermented Beverages

The fermentation workflow was adapted to the specific characteristics of the raw materials. High-power homogenisation using Ultraturrax equipment was avoided because the high WHC of the samples was expected to create stable emulsions. Instead, low shear homogenisation performed manually for 3 min was employed to maintain the integrity of the samples. This high water retention also limited sample extraction for certain analyses due to the low availability of free water, particularly in the VPF and ROF samples, this being consistent with their measured water-holding capacities.

#### 3.1.2. Screening of Fermented Beverages with *Lactiplantibacillus plantarum* and *Bacillus subtilis* of Vegetable By-Products

**Figure 2:**
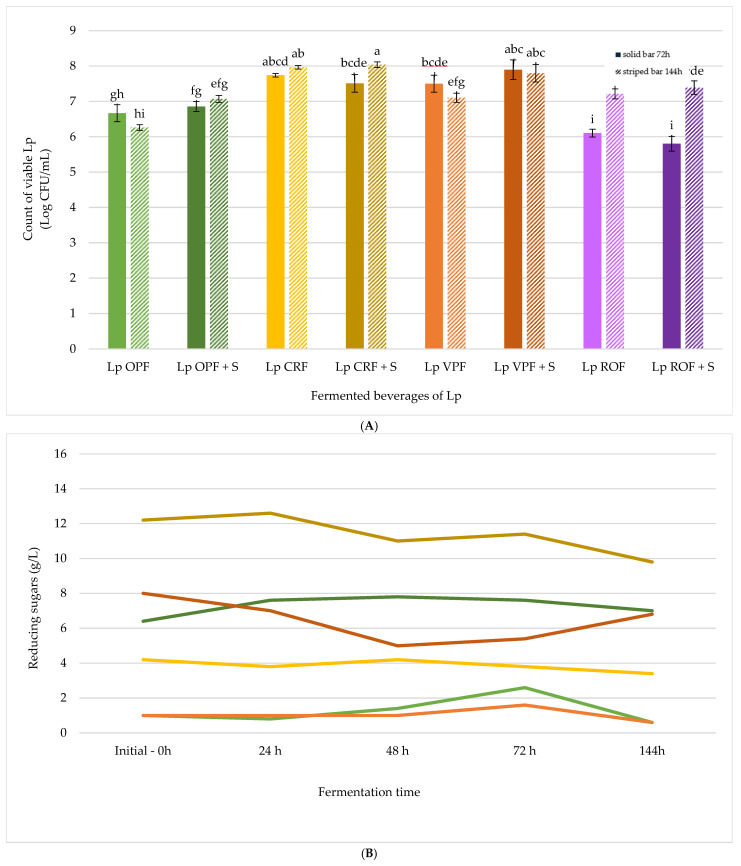
(**A**) Graphical depiction of the results of the viable cell counts (log CFU/mL) of *Lp* in fermented beverages after 72 h (solid bars) and 144 h (striped bars). (**B**) Graphic depiction of the reducing sugar content (g/L) during the fermenting of beverages of *Lp* at 0, 24, 48, 72, and 144 h. Samples: 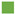 Lp OPF, 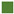 Lp OPF + S, 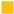 Lp CRF, 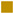 Lp CRF + S, 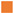 Lp VPF, 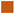 Lp VPF + S, 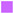 Lp ROF, and 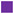 Lp ROF + S. Different letters indicate statistically significant differences between fermented beverages at *p* < 0.05.

The fermentation potential of the four fibre extracts—OPF, VPF, CRF, and ROF—was evaluated using *Lp* as the inoculum. The initial inoculum concentration was adjusted to an optical density (OD600) of 0.6, corresponding to approximately 7.75 CFU/mL. Upon inoculation (1% *v*/*v*), the starting microbial concentration in the fermentation media was approximately 5.75 log CFU/mL (Figure 2A).

As shown in Figure 2A, *Lp* exhibited growth in all formulations, with a significantly enhanced proliferation in samples supplemented with table sugar (10 g/L). This confirms the role of added sugars as an accessible carbon source that supports microbial metabolism. Among the unsweetened samples, CRF and VPF supported moderate growth, while ROF showed minimal microbial proliferation, likely due to its lower initial pH (Table 3), which may have inhibited bacterial activity. Interestingly, the second stage of fermentation (additional 72 h in sterile bottles) further promoted microbial growth in some samples, particularly CRF and ROF, suggesting that extended fermentation under modified conditions may help overcome initial inhibitory factors.

Figure 2B illustrates the evolution of the reducing sugars during fermentation. As expected, sugar-supplemented samples exhibited higher initial sugar concentrations and a more noticeable decline over time, reflecting microbial consumption. CRF, even without added sugar, showed a relatively high natural sugar content, which may explain its ability to support microbial growth. In contrast, OPF and VPF had lower initial sugar levels, correlating with more modest microbial activity in the absence of supplementation. Only six fermented beverages appear in Figure 2B, since ROF was excluded due to its high viscosity, which prevented the reliable measurement of reducing sugar content. This limitation is consistent with its exceptionally high WHC, as reported in Table 2, which reduces free water availability and justifies its exclusion from the scaling experiments.

The pH values decreased progressively throughout the two-phase fermentation process (Table 3), which is consistent with the production of organic acids by *Lp* [20]. The extent of acidification varied between the fibre-rich extracts. This was influenced by their initial pH and fermentable sugar content. In most cases, the final pH values fell below 5.0, thus indicating a microbiologically stable environment suitable for food safety. The most pronounced pH reductions were observed in the sugar-supplemented formulations, particularly Lp VPF + S and Lp OPF + S.

A final pH below 4.5, observed in most *Lp*-fermented fibre-rich extracts, ensures microbial safety, a critical parameter for commercial viability. These results are consistent with previous studies [9], which show that LAB fermentation produces organic acids and antimicrobial compounds, thus stabilising non-dairy fermented beverages.

Furthermore, Lp-fermented products are also known to deliver health-promoting metabolites (e.g., hydroxytyrosol from olive by-products). This reinforces the nutritional relevance of using substrates like OPF [9].

The sensory evaluation revealed that *Lp*-fermented samples generally exhibited balanced and mild flavour profiles. The OPF and VPF beverages retained subtle, pleasant notes reminiscent of their original plant matrices—olive and vine prunings, respectively. In contrast, the CRF samples showed persistent bitterness. Sweetened versions were perceived as more palatable across all matrices, enhancing flavour and reducing off-notes.

The concept of valorising fibre-rich agro-industrial by-products via fermentation is supported by several recent studies. Benucci et al. [20] emphasised the circular economic potential of such approaches, citing enhanced polyphenol bioavailability and improved antioxidant properties when food wastes are transformed into functional beverages. Additionally, Kaur et al. [3] highlighted that fermentation processes using LAB—especially *Lp* —can mitigate bitterness and enhance sensory profiles of fibrous waste matrices, consistent with our sensory panel results for OPF and VPF. The fermentation performance of *Lp*, especially in sugar-supplemented formulations, aligns with prior studies highlighting its robust growth and acidification capabilities across plant-based substrates. Lee et al. [21] observed enhanced functional and sensory properties in a plant-based beverage co-fermented with *Lp*, corroborating our findings of improved flavour and sugar metabolism, particularly in the CRF and VPF samples.

The enzymatic activity of *Lp*, including the decarboxylation of phenolic acids—such as hydroxycinnamic acid metabolism—may have contributed to the mild sensory profile and observed pH reductions, as previously detailed by [14]. These transformations are particularly relevant for polyphenol-extracted residues like OPF and VPF, which retain trace bioactives after subcritical water extraction.

The differential support for microbial growth observed in ROF and CRF is likely attributable to both fibre architecture and residual sugar content. CRF supported higher *Lp* proliferation, which aligns with the findings of [22], who demonstrated that substrates containing simple sugars and prebiotic compounds such as inulin and fructooligosaccharides significantly enhance the viability and stability of *Lp* during fermentation. Furthermore, the presence of water-soluble carbohydrates such as fructans and pectins—particularly in CRF—is known to enhance microbial proliferation and metabolite production [13], which explains CRF’s suitability even in unsweetened formulations.

#### 3.1.3. Screening of Fermented Beverages with *Bacillus subtilis*

**Figure 3:**
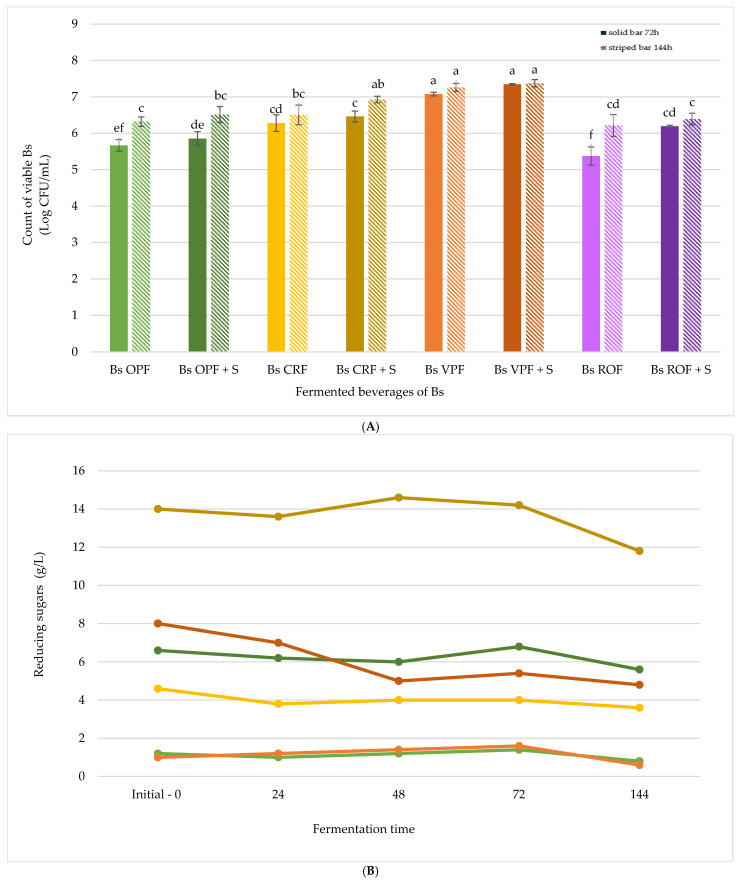
(**A**) Graphic depiction of viable cell counts (log CFU/mL) of Bacillus subtilis in fermented beverages after 72 h (solid bars) and 144 h (striped bars). (**B**) Graphical depiction of the reducing sugar content (g/L) in fermented beverages of Bacillus subtilis at 0, 24, 48, 72, and 144 h. Samples: 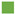 Bs OPF, 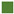 Bs OPF + S, 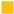 Bs CRF, 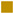 Bs CRF + S, 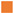 Bs VPF, 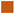 Bs VPF + S, 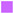 Bs ROF, and 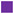 Bs ROF + S. Different letters indicate statistically significant differences between fermented beverages at *p* < 0.05.

The fermentation performance of *Bs* was evaluated using the same fibre extracts (OPF, VPF, CRF, and ROF) under identical conditions to those used for *Lp*. The initial inoculum was adjusted to OD600 = 0.6, corresponding to approximately 7.98 log CFU/mL. After inoculation (1%; *v*/*v*), the starting concentration in the fermentation media was approximately 5.98 log CFU/mL (Figure 3A).

As shown in Figure 3A, *Bs* exhibited limited growth in most formulations, except for VPF, where a notable increase in viable cell counts was observed. In general, sugar supplementation (10 g/L) enhanced microbial proliferation across all samples, confirming its role as a fermentable carbon source. However, in the OPF, CRF, and ROF samples, microbial growth was minimal, suggesting that these substrates may lack nutrients or may contain inhibitory compounds that affect *Bs* viability. The second fermentation phase (additional 72 h) resulted in only a slightly increase in viable counts, particularly in sugar-supplemented formulations, but overall microbial growth remained lower than that observed with *Lp*.

Figure 3B shows the evolution of reducing sugars during fermentation. As expected, sugar-supplemented samples had higher initial sugar concentrations and exhibited a more pronounced decrease over time. In contrast, unsupplemented formulations showed minimal changes, with OPF and VPF maintaining very low sugar levels throughout the process. CRF, which naturally contained more sugars, showed moderate reductions, particularly in the sugar-supplemented variant. Similarly to *Lp*, in Figure 2B, only six fermented beverages are shown. ROF was not included due to its viscosity, which hindered accurate sugar measurements. As previously mentioned, this behaviour can be attributed to its high WHC (Table 2), and therefore this extract was not considered for scale-up.

The pH values of *Bs* fermentation are presented in Table 4. Unlike the acidification observed with *Lp* (Table 3), the pH changes in *Bs* fermentations were minimal. Slight decreases were noted in the CRF, ROF, and VPF samples, particularly in sugar-supplemented formulations. In the OPF samples, the pH remained nearly constant throughout the process. These results suggest that *Bs* did not produce significant amounts of organic acids under the tested conditions, which may be linked to its limited growth and metabolic activity.

Focus group analysis indicated that *Bs* fermentation resulted in more complex and occasionally undesirable sensory outcomes. The OPF and CRF samples were broadly similar to their *Lp* counterparts—with slightly enhanced lactic and umami-like notes. Notably, the VPF samples developed off-flavours characterised by participants as “overly fermented” or “spoiled,” possibly due to specific metabolic by-products generated by *Bs* in this fibre matrix.

#### 3.1.4. Comparative Evaluation of *Lactiplantibacillus plantarum* and *Bacillus subtilis*

The comparative analysis of the two microbial strains, *Lp* and *Bs*, revealed clear differences in fermentation performance, guiding the selection of conditions for scale-up formulations. *Lp* consistently demonstrated superior growth across all substrates, particularly in sugar-supplemented formulations, with final viable counts exceeding 8 log CFU/mL in several cases. This strain also induced a more pronounced acidification of the medium, with pH values dropping below 4.5, contributing to microbial stability and food safety. In contrast, *Bs* exhibited limited growth in most formulations, except for VPF + S, showing minimal acidification. The lower metabolic activity of *Bs* under the tested conditions resulted in less consistent fermentation outcomes and, in some cases, the development of undesirable sensory attributes—particularly in VPF-based samples. Our findings align with the established literature: Lp is known to reduce bitterness and enhance umami through aldehyde-to-alcohol conversion, contributing to a mild and balanced sensory profile [23]. Conversely, *Bs* fermentations often produce off-flavours—such as rancid, spoiled, or overly fermented notes. These are attributed to volatile compounds like isovaleric, isobutyric, and 2-methylbutyric acids [24]. Our results align with previous studies on sensory modulation in plant-based beverages. For instance, Yan et al. [25] reported that *L. fermentum grx08* reduced off-flavours, especially bitterness, and enhanced fruity aromas and mild acidity in a compound a plant-based compound beverage, supporting our findings in the OPF and VPF fermentations. Based on these findings, *Lp* was selected as the preferred microorganism for scale-up due to its robust growth, effective acidification, and more favourable sensory profile. However, *Bs* was retained in a selected formulation (OPF + S) where its performance was acceptable, to explore its potential in combination or under optimised conditions in future studies.

### 3.2. Scale-Up of Fermented Beverages

Based on the screening results, three formulations were selected for scaling up: (1) Lp OPF + S, (2) Lp VPF + S, and (3) Bs OPF + S. These samples were chosen due to their favourable microbial growth, pH reduction, and acceptable sensory characteristics. The scaling up was performed at a 1:5 ratio, maintaining the same proportions of fibre, water, sugar, and inoculum as in the screening experiments.

**Figure 4:**
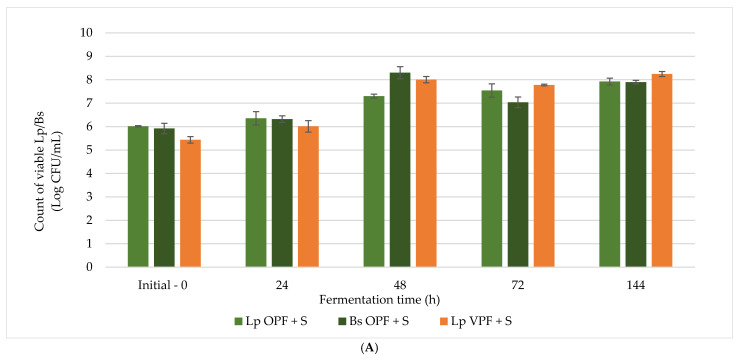

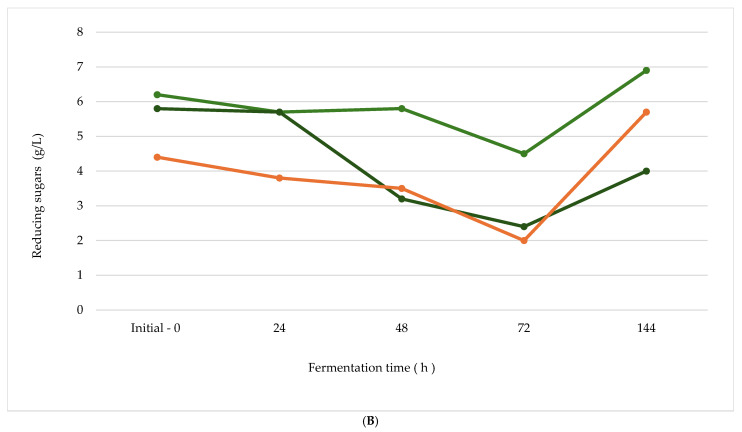
(**A**) Graphic depiction of the results of viable cell counts (log CFU/mL) in 3 fermented beverages during the first and second fermentations. (**B**) Graphic depiction of the reducing sugar content (g/L) in fermented beverages at 0, 24, 48, 72, and 144 h on the scaled-up fermented beverages. Samples 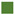 Lp OPF + S, 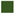 Bs OPF + S, and 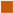 Lp VPF + S.

As shown in Figure 4A, microbial growth was successfully maintained or enhanced during scaling-up. *Lp* reached final concentrations above 9.5 log CFU/mL in both OPF + S and VPF + S formulations, indicating robust proliferation under scaled conditions. *Bs* also showed improved growth in the OPF + S formulation, reaching similar final counts. These results confirm that the selected conditions supported microbial viability and activity in larger volumes.

The pH values followed a similar trend to the screening phase (Table 5), with a gradual decrease throughout the fermentation process. The most pronounced acidification was observed in Lp VPF + S, where the pH dropped below 4.0 by the end of the second phase of fermentation (144 h). This indicates strong metabolic activity and suggests a stable, microbiologically safe product. Bs OPF + S also showed a moderate pH reduction, although less pronounced than the *Lp* formulations, reflecting its comparatively lower metabolic activity.

Reducing sugar levels decreased progressively during fermentation (Figure 4B), particularly in sugar-supplemented formulations. The most significant reductions were observed in Lp VPF + S and Bs CRF + S, indicating active sugar metabolism. Interestingly, sugar levels increased slightly in some samples during the second fermentation, possibly due to the breakdown of complex carbohydrates into simpler sugars.

The scaled-up experiments confirmed the feasibility of producing fermented beverages from fibre-rich by-products under control conditions. *Lp* demonstrated a good performance in terms of microbial growth, acidification, and sensory quality, making it the preferred strain for further development. The selected formulations maintained their functional and microbiological properties at larger volumes, supporting their potential for industrial application.

### 3.3. Antioxidant Potential of Scale-Up Fermented Beverages

Fermentation is recognised as an effective strategy to enhance the antioxidant properties of plant-based and lignocellulosic matrices through microbial biotransformation of bound phenolics, enzymatic hydrolysis of structural polysaccharides, and the generation of bioactive metabolites [2,3]. In the present study, the scale-up fermentation of fibre extracts (OPF, VPF) demonstrated that microbial metabolism significantly influenced both total phenolic content (TPC) and antioxidant capacity (ORAC).

The total phenolic content (TPC) and antioxidant capacity (ORAC) of the scale-up fermented beverages at 72 h and 144 h are shown in Table 6. To isolate the specific effect of microbial fermentation, control samples were prepared under identical incubation conditions but without microbial inoculation. These uninoculated controls (OPF: 169.8 mg GAE/L, 607.5 µmol TE/L; VPF: 2.03 mg GAE/L, 119.5 µmol TE/L) showed only minor variations over a 144 h period of fermentation, confirming that the observed changes in the inoculated samples were primarily driven by microbial metabolism rather than passive solubilisation or non-enzymatic hydrolysis.

Fermentation with Lp markedly increased ORAC values, particularly in the VPF-based substrate. After 72 h, intermediate antioxidant levels were observed (Lp OPF + S: ~710 µmol TE/L; Lp VPF + S: ~251 µmol TE/L), which further increased at 144 h (Lp OPF + S: ~750 µmol TE/L; Lp VPF + S: ~267 µmol TE/L). These trends indicate progressive enzymatic hydrolysis of conjugated phenolics and accumulation of low-molecular-weight antioxidant metabolites. In contrast, Bs fermentation in OPF + S resulted in a smaller ORAC improvement and a reduction in TPC after 144 h, likely due to limited hydrolytic enzyme activity and partial oxidative degradation of phenolics. These results highlight the importance of strain-specific enzymatic capabilities in determining the antioxidant potential of lignocellulosic substrates [26].

The observed enhancement in antioxidant capacity can be attributed to a synergistic interplay among residual phenolics, enzymatic biotransformation, and microbial metabolite formation. Enzymes such as β-glucosidase, tannase, and feruloyl esterase catalyse the cleavage of glycosidic or ester bonds, releasing phenolic acids and aglycones with higher radical-scavenging potential [27,28]. Simultaneously, microbial metabolites—including organic acids, peptides, and exopolysaccharides—can contribute additional antioxidant activity through radical scavenging and metal chelation [29,30]

Matrix composition also plays a critical role in modulating antioxidant activity. OPF, with a relatively higher content of soluble fibre, might favour faster microbial colonisation and metabolite accumulation, whereas VPF, characterised by higher insoluble fibre, supported gradual enzymatic hydrolysis and sustained phenolic release over extended fermentation. The presence of supplemental sugars enhanced co-metabolic pathways, providing energy for enzyme synthesis and secondary metabolite production.

These findings are consistent with earlier studies reporting improved antioxidant capacity in fermented lignocellulosic residues. For example, fermentation of wheat bran and rice husk with *Lp* enhanced radical-scavenging activity through ferulic acid release and formation of antioxidant peptides and polysaccharides [30]. Similarly, microbial fermentation of flaxseed cake and soybean residues increased ORAC values via the bioconversion of fibre-bound phenolics and synthesis of bioactive metabolites [31].

The inclusion of incubated controls confirms that these increases are primarily microbially driven, rather than due to passive chemical changes. The extent of ORAC enhancement correlates positively with initial residual phenolic content, as phenolic-rich substrates (VPF) exhibit greater absolute gains than low-phenolic matrices (OPF). Although final ORAC values remained below those of polyphenol-rich botanical beverages, controlled fermentation effectively valorises pruning-derived lignocellulosic by-products into functional beverages with measurable radical-scavenging capacity.

In conclusion, the antioxidant potential of scale-up fermented beverages results from the combined effects of enzymatic hydrolysis, phenolic transformation, and microbial metabolite formation. The fermentation of subcritical-water-treated pruning residues therefore represents a promising valorisation approach aligned with circular bioeconomy principles, enabling the conversion of low-value lignocellulosic materials into functional beverages with potential health-promoting properties.

## 4. Conclusions

Fermented beverages were successfully developed using fibre-rich extracts derived from agro-industrial by-products. Although the water-holding capacity of some fibre extracts was excessive, rendering them unsuitable for producing beverage, these may have potential for other functional food applications—such as texturising agents or prebiotic-rich solids.

Among the microbial strains tested, *Lactiplantibacillus plantarum* consistently outperformed *Bacillus subtilis* in terms of growth acidification and sensory properties. Supplementation with sugar consistently enhanced microbial proliferation and improved the overall palatability of the beverages, highlighting the importance of optimising the carbon source in fermentation-based formulations. Notably, the final products exhibited low pH values (<4.5). These ensures microbial safety and stability without the need for additional preservatives.

Three optimised formulations were successfully scaled up—Lp OPF + S, Lp VPF + S, and Bs OPF + S—maintaining or improving microbial viability, acidification levels, and fermentation performance. These results confirm the feasibility of industrial-scale production of functional fermented beverages using fibre-rich food by-products. In contrast, the excessive water-holding capacity of ROF fractions limits their use in beverages. However, they may still represent valuable ingredients for other applications, such as texturising agents or prebiotic-rich solids.

It is important to highlight that Lp fermentation markedly enhanced antioxidant potential, with ORAC values increasing up to ~750 µmol Trolox equivalents/L and TPC up to ~18 mg GAE/L. This improvement reflects the microbial biotransformation of bound phenolics and accumulation of antioxidant metabolites. Although microbial co-cultures were not investigated in this study, future research could explore synergistic fermentations or metabolic complementation between strains to further enhance antioxidant potential and functional compound release further.

Overall, this study highlights the valorisation of agri-food residues through microbial fermentation, contributing to models of the circular economy and offering a sustainable pathway for the development of next-generation functional beverages.

## Figures and Tables

**Table 1:** Experimental design and sample nomenclature of the screening experiments.

Microorganism Inoculated	*Lactiplantibacillus plantarum* (Lp)		*Bacillus subtilis* (Bs)	
	No Addition	Sugar (S): 10 g/L	No Addition	Sugar (S): 10 g/L
OPF	Lp OPF	Lp OPF + S	Bs OPF	Bs OPF + S
VPF	Lp VPF	Lp VPF + S	Bs VPF	Bs VPF + S
CRF	Lp CRF	Lp CRF + S	Bs CRF	Bs CRF + S
ROF	Lp ROF	Lp ROF + S	Bs ROF	Bs ROF + S

Olive pruning fibre (OPF), vineyard pruning fibre (VPF), chicory root fibre (CRF), and red onion fibre (ROF).

**Table 2:** Results of the characterisation of the initial fibre-rich extracts: moisture (g/100 g), water-holding capacity (%), soluble and insoluble fibres (%).

Source	Moisture (g/100 g)	Water-Holding Capacity (%)	Soluble Fibre (%)	Insoluble Fibre (%)
OPF	7.75 ± 0.23 a	285.43 ± 2.38 d	2.70 ± 0.50 a	83.20 ± 1.70 a
VPF	5.19 ± 0.16 b	388.06 ± 8.11 b	0.50 ± 0.10 b	90.50 ± 0.90 b
CRF	4.35 ± 0.20 b	357.37 ± 3.09 c	0.50 ± 0.10 b	55.90 ± 0.50 c
ROF	8.06 ± 0.22 a	456.65 ± 2.52 a	1.50 ± 0.30 c	73.30 ± 0.90 d

Olive pruning fibre (OPF), vineyard pruning fibre (VPF), chicory root fibre (CRF), and red onion fibre (ROF). Different letters indicate statistically significant differences between samples at *p* < 0.05.

**Table 3:** Results of the measurement of the pH of the fermented beverages of *Lp* at the fermentation times of initial, 24, 48, 72 h, and finally, 144 h.

pH	Initial (0 h)	Fermentation			
		24 h	48 h	72 h	144 h
Lp OPF	4.96	4.85	4.77	4.60	4.43
Lp OPF + S	4.95	4.83	4.72	4.55	4.27
Lp VPF	4.72	4.71	4.64	4.42	4.37
Lp VPF + S	4.71	4.66	4.52	4.34	4.22
Lp CRF	5.54	5.43	5.35	5.27	5.01
Lp CRF + S	5.55	5.33	5.05	5.03	4.90
Lp ROF	4.49	4.50	4.43	4.46	4.14
Lp ROF + S	4.50	4.35	4.33	4.33	4.07

**Table 4:** Results of the measurement of the pH of the fermented beverages of *Bacillus subtilis* (*Bs*) at the fermentation times of initial, 24, 48, 72 h, and finally, 144 h.

pH	Initial (0 h)	Fermentation			
		24 h	48 h	72 h	144 h
Bs OPF	4.98	5.02	4.96	5.05	4.99
Bs OPF + S	4.97	5.01	4.93	5.04	4.91
Bs VPF	4.70	4.70	4.70	4.72	4.62
Bs VPF + S	4.68	4.66	4.65	4.60	4.51
Bs CRF	5.51	5.66	5.69	5.45	5.32
Bs CRF + S	5.48	5.65	5.65	5.46	5.29
Bs ROF	4.50	4.51	4.54	4.50	4.35
Bs ROF + S	4.52	4.48	4.46	4.46	4.31

**Table 5:** Results of the measurement of pH of the scaled-up fermented beverages at the fermentation times of initial, 24, 48, 72 h, and finally, 144 h.

pH	Initial (0 h)	Fermentation			
		24 h	48 h	72 h	144 h
Lp OPF + S	5.05	4.97	4.91	4.87	4.23
Bs OPF + S	5.04	4.98	4.89	4.76	4.35
Lp VPF + S	4.77	4.68	4.32	3.98	3.91

**Table 6:** Results of the measurement of total polyphenol content (TPC) and antioxidant activity (ORAC) of the scaled-up fermented beverages at 72 and 144 h of fermentation.

Scale-Up Fermented Beverage	Total Phenolic Content (mg GAE/L)			ORAC (µmol Trolox/L)		
	Control	72 h	144 h	Control	72 h	144 h
Bs OPF + S	169.80 ± 1.98 b	201.98 ± 3.42 a	139.68 ± 3.85 c	607.54 ± 7.07 a	268.05 ± 4.91 c	309.45 ± 6.79 b
Lp OPF + S	169.80 ± 1.98 a	5.70 ± 0.99 c	17.88 ± 0.74 b	607.54 ± 7.07 b	709.25 ± 5.08 a	750.08 ± 22.37 a
Lp VPF + S	2.03 ± 0.52 a	3.40 ± 1.41 a	3.33 ± 2.36 a	119.54 ± 12.73 b	250.75 ± 5.46 a	267.08 ± 7.78 a

Different letters indicate statistically significant differences between fermented beverages at *p* < 0.05.

## Data Availability

The original contributions presented in this study are included in the article. Further inquiries can be directed to the corresponding author(s). Experimental data are available at: https://doi.org/10.34810/data2692.
